# Human Tripod

**Published:** 1847-08

**Authors:** 


5. *Human Tripod.*—A malformed child, so called from its presenting the appearance of three legs, is described and figured in the last volume of the "Medico-Chirurgical Transactions," by Mr. Acton. The child is represented as being lively and healthy, and perfectly formed above the umbilicus. Below this point, and to the right and left of the mesial line are two distinct penes, of normal direction and size. Each is provided with a scrotum, the outer half of each containing a testicle. Between and behind the legs is seen another limb, or rather two limbs united together throughout their entire length. The anus occupies its usual situation, and the functions of the bowels are duly performed. Below this the the thigh of the compound limb equals in size the buttocks of a young child. Urine passed at the same time from both penes. Mr. Acton mooted the question of the removal of this supplementary limb, which he considered a feasible operation.—*Medico-Chirurg. Trans.*, vol. xxix., *ibid.*

